# Exploration of causal relationship between shoulder impingement syndrome and rotator cuff injury: a bidirectional mendelian randomization

**DOI:** 10.1186/s12891-024-07556-1

**Published:** 2024-08-19

**Authors:** Li Liu, Fan Yang, Ying Liao, Hui Deng, Dongsheng Le, Chao Zhang, Mailin Zhao, Pingsheng Liao, Yingping Liang

**Affiliations:** 1 Department of Pain Medicine, the Second Affiliated Hospital, Jiangxi Medical College, Nanchang University, No. 1 Mingde Street, Donghu District, Nanchang, P.R. China; 2 Beimen Street Community Health Service Center, Jizhou District, Jian, China; 3 Department of Orthopedics, the Second Affiliated Hospital, Jiangxi Medical College, Nanchang University, Nanchang, China

**Keywords:** Shoulder impingement syndrome, Rotator cuff injury, Mendelian randomization, Causal relationship, Genome wide association study

## Abstract

**Backgroup:**

The pathogenesis of shoulder impingement syndrome (SIS) is still unclear, and its questionable causal relationship with rotator cuff (RC) injury has led to confusion in treatment. The purpose of this study was to explore the bidirectional causal relationship between SIS and RC injury.

**Methods:**

SIS and RC injury datasets downloaded from the IEU Open GWAS project and GWAS catalog databases. Inverse variance weighted (IVW), MR Egger, Weighted median, and Weighted mode were used in this Mendelian randomization (MR) analysis. Cochran’s Q test, leave-one-out, and funnel plot method were used to evaluate heterogeneity between single nucleotide polymorphisms (SNPs). MR-Egger regression was used to test the horizontal pleiotropy of this study.

**Results:**

The IVW method (OR = 1.189, *P* = 0.0059) suggest the putative causal effect of RC injury on SIS. The results of MR Egger method (OR = 1.236, *P* = 0.2013), weighted median method (OR = 1.097, *P* = 0.2428) and weighted mode method (OR = 1.013, *P* = 0.930) showed no statistically significant (OR = 1.069071, *P* = 0.6173). Heterogeneity test and horizontal pleiotropy analysis suggested that there was no significant heterogeneity and horizontal pleiotropy in the results of this MR analysis. The reverse MR analysis showed heterogeneity, and the conclusion needs to be further explored.

**Conclusions:**

The results of MR analysis support that RC injury may be causally associated with SIS.

**Supplementary Information:**

The online version contains supplementary material available at 10.1186/s12891-024-07556-1.

## Introduction

Shoulder pain is one of the most common types of pain, with a prevalence of between 7% and 30% [1–4]. Shoulder impingement syndrome (SIS) is one of the most common causes of shoulder pain, which could not be fully explained so far [5–7]. Injury of rotator cuff (RC) are often found to occur at the same time as SIS [8]. Some studies have suggested that the injury of RC may lead to a decrease in joint stability, which may be the causes of SIS [9–11]. Other studies suggest that SIS can damage the tendons around the shoulder, leading to RC injury and shoulder pain [12–15]. These are called the intrinsic and extrinsic mechanisms. Although the theory of intrinsic mechanism has become more and more popular in recent years [16, 17], there is still a lack of evidence to prove the relationship between the two theories.

Most of the early studies support the extrinsic theory, which holds that different shapes of acromion are important cause of SIS [9], and surgical decompression is very important, which can prevent the progression of the disease and avoid further injury of RC [18–20]. However, some studies have found that decompression surgery will not improve the prognosis of patients with SIS [21–23]. Some scholars have proposed that the intrinsic theory may be the cause of SIS [24]. The conflict between these two theories poses a major challenge in the management of patients with SIS, which still cannot be fully explained [25]. Therefore, it is important to explore the causal relationship between RC injury and SIS, which may help guide the diagnosis and treatment of patients with shoulder pain in the future.

**Fig. 1:**
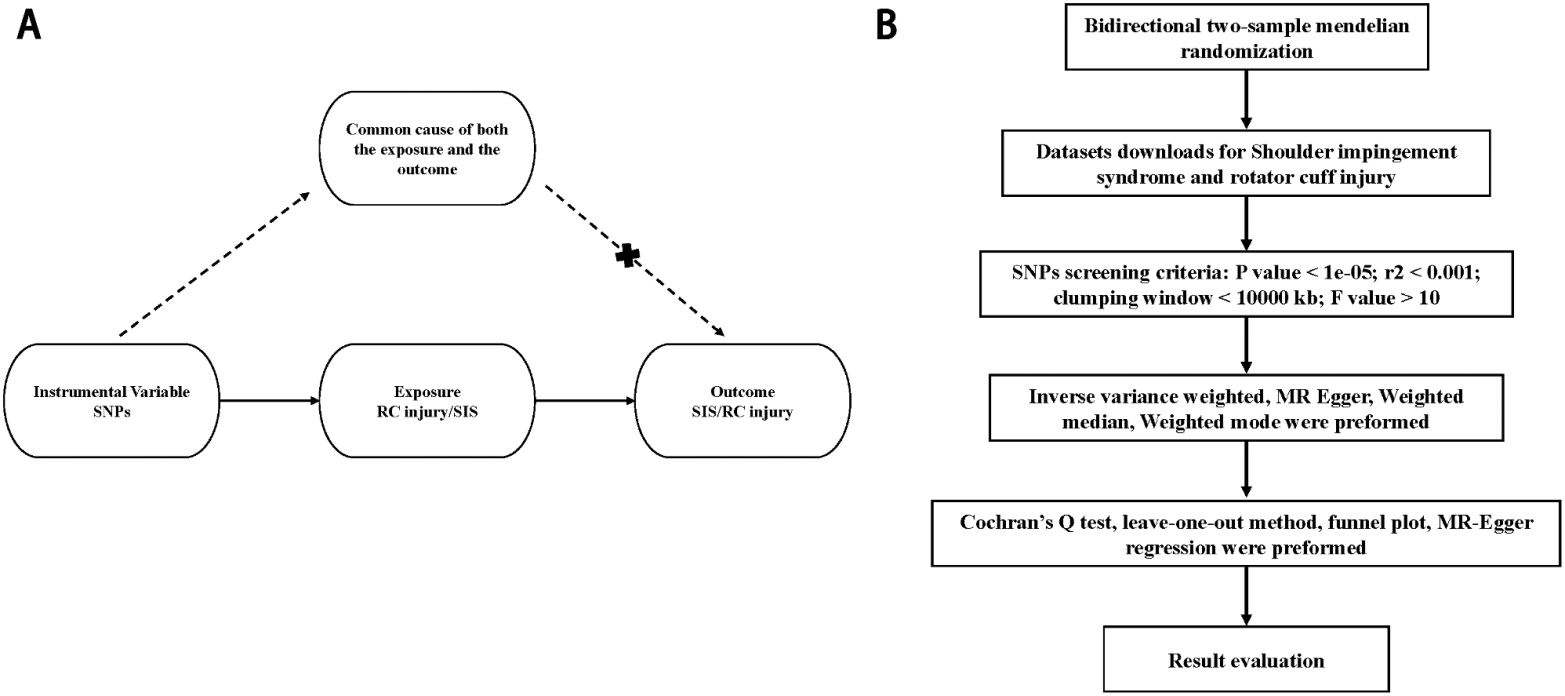
The process of Mendelian Randomization. (**A**) The hypothesis principle of Mendelian Randomization; (**B**) The research workflow of this paper

Based on the rapid development of Genome wide association study (GWAS) [26], Mendelian randomization (MR) analysis has been used to explore the causal relationship between exposure factors and outcome factors in recent years [27, 28]. MR uses the characteristics of germline genetic variation (usually in the form of single nucleotide polymorphism (SNP)) that are closely related to presumptive exposure as instrumental variables (IVs) to test and estimate the causal effect of exposure on outcome (Fig. 1A). The operation of MR is based on three assumptions about IVs, namely: correlation assumption (genetic variation used as IVs are related to exposure); exclusion restriction assumption (genetic variation and target outcome have no common cause); independence assumption (there is no independent causal pathway between genetic variation and outcome except through exposure), and using the principle of Mendel’s law of inheritance, MR analysis can play a role similar to randomized controlled test (RCT) to explore the causal relationship between exposure factors and outcome factors. The aim of this study was to investigate the bidirectional causal relationship between RC injury and SIS by using MR analysis.

## Methods

### Data sources

Data for this study were obtained from the IEU Open GWAS project (https://gwas.mrcieu.ac.uk/) and GWAS catalog [29] (https://www.ebi.ac.uk/gwas/). Two datasets were used in this study: one for rotator cuff injury (GWAS id: ebi-a-GCST90044700) and one for shoulder impingement (GWAS id: ebi-a-GCST90014054). The rotator cuff injury dataset contains 5701 case data and 406,310 control data, and the dataset contains 16,110,542 SNPs. The shoulder impingement syndrome dataset contains 3864 case data and 208,258 control data, and the dataset contains 16,415,789 SNPs. Patients included in the study for both datasets were European.

### Selection of instrumental variables and mendelian randomization analysis methods

We selected SNPs with strong correlations with exposure factors as instrumental variables (P value < 1e-05), and we also set conditions for screening these SNPs to eliminate the effect of linkage disequilibrium (LD) on this study (r^2^ < 0.001 within a clumping window of 10,000 kb). F statistic was also used to screen strongly correlated IV, the IVs included in the study must also conform to F value > 10. With the help of the above methods, it is proved that the acquisition of SNPs with strong correlation with exposure factors accords with the correlation hypothesis of MR. If a particular exposure SNP is not present in an outcome dataset, proxy SNPs were used instead through LD tagging. Inverse variance weighted (IVW), MR Egger, Weighted median, Weighted mode methods were used for MR analysis in this study **(**Fig. 1B**)**. The IVW method uses a meta-analysis method to analyze the Wald ratio of each SNP included in the study [30]. But the premise of the IVW method is that all contained SNP must be valid variables, while the MR Egger method can still work even if the SNP is an invalid variable. The MR Egger method takes into account the existence of the intercept term in the regression analysis, so it can still be effective when the SNP has multiple effects [31]. The weighted median method works when the valid variable is not less than 50% [32]. The weighted mode method can reduce the errors caused by the deviation of some genetic variation estimation results [31]. All MR analyses for this study were performed using the R software (R version: 4.0.3), the “TwoSampleMR” package was used for analysis.

### Heterogeneity analysis and horizontal pleiotropy analysis

Sensitivity analysis was used to assess heterogeneity between instrumental variables. Cochran’s Q test [33], leave-one-out method [34], and funnel plot method were used to evaluate heterogeneity between SNPs. Setting the Cochran’s Q test P value < 0.05 is considered heterogeneous between SNPs. The leave-one-out method analyses the effect of individual SNPs on the final results by removing them one by one, while the funnel plot method determines the heterogeneity of the study from the distribution of SNPs on the plot. Horizontal pleiotropy can seriously affect the results of MR analyses, which we tested using MR-Egger regression [35]. Setting directionality P value < 0.05 is having horizontal pleiotropy, then the conclusion is unreliable **(**Fig. 1B**)**. Odds ratios (ORs) values and 95% confidence intervals (CIs) were used to describe the MR results. These methods are helpful to evaluate the validity of the results and are consistent with the independence assumption and the exclusion restriction assumption.

## Results

### Mendelian randomization results of rotator cuff injury on the risk of shoulder impingement syndrome

**Fig. 2:**
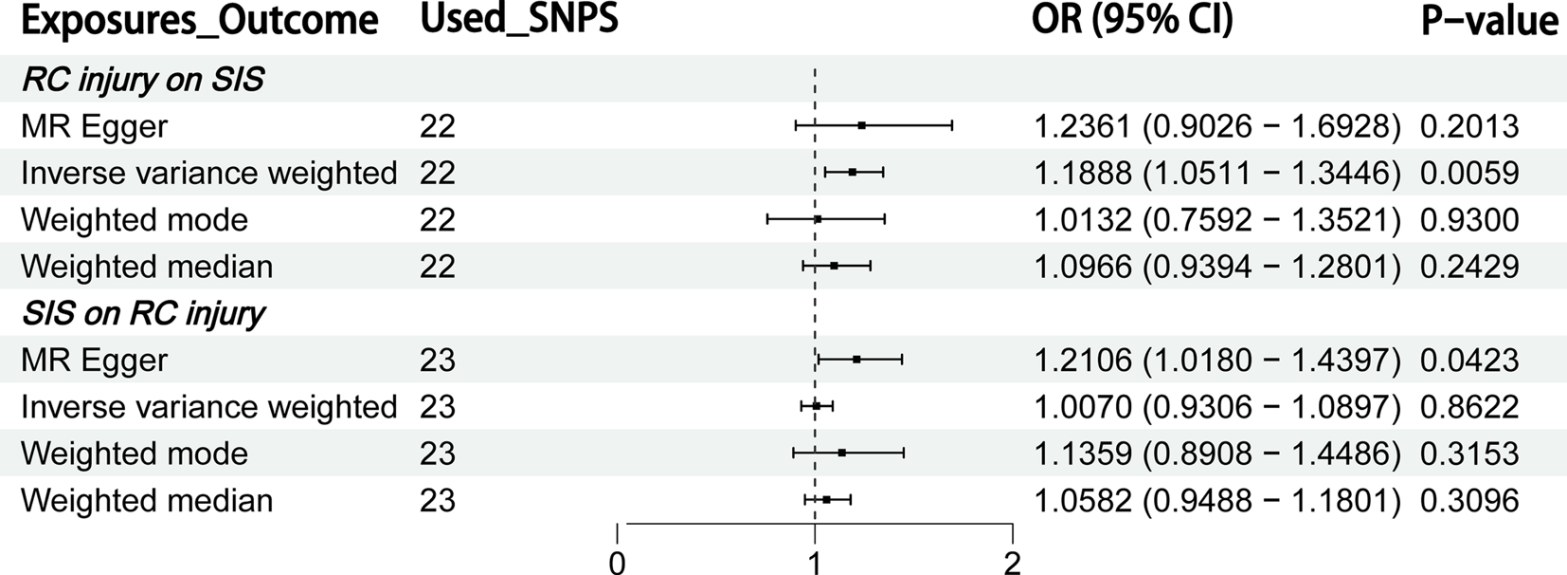
The result of Bidirectional Mendelian Randomization

**Fig. 3:**
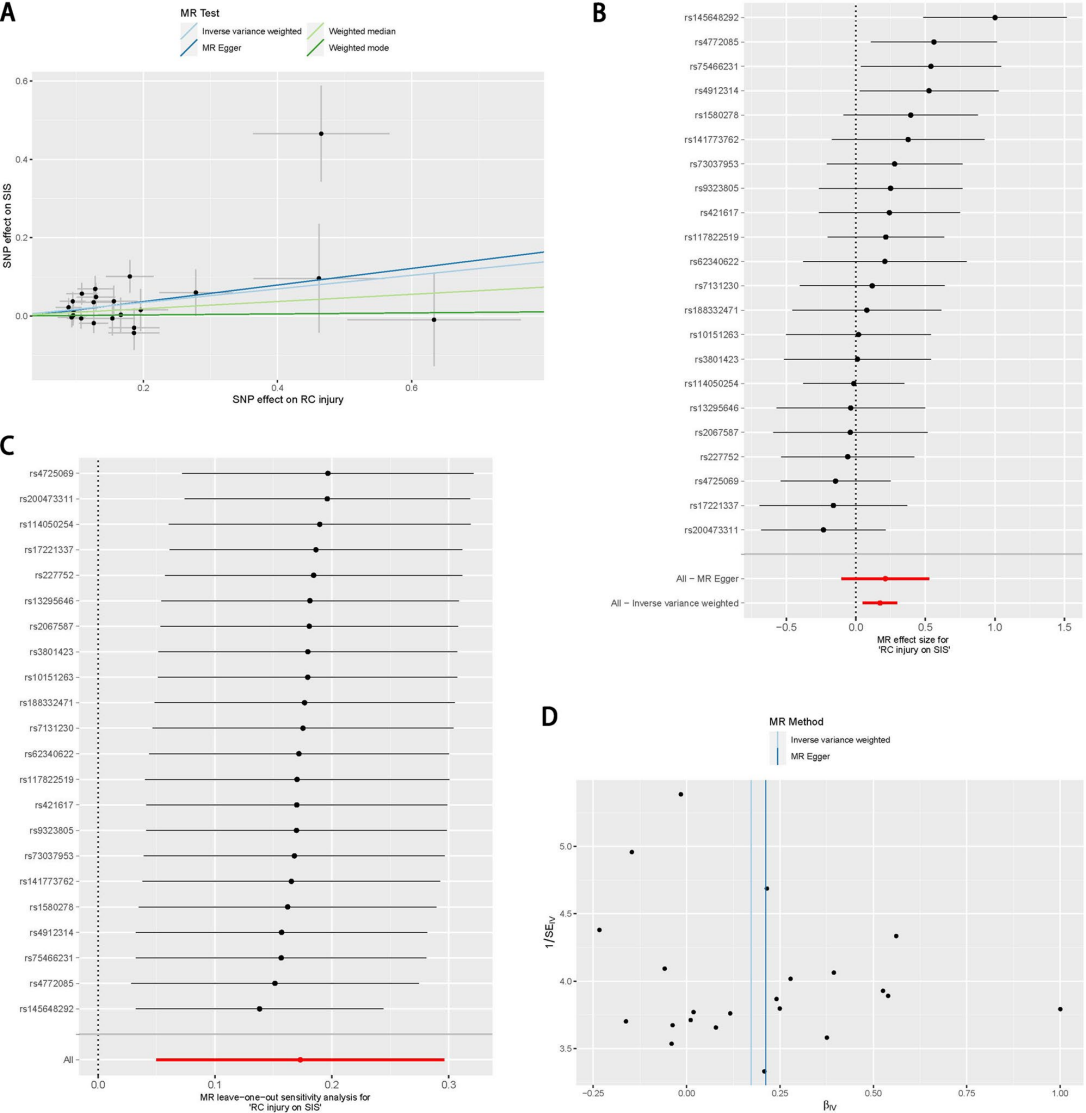
The putative causal effect of rotator cuff injury on shoulder impingement syndrome. (**A**) Scatter plots of genetic associations with rotator cuff injury against the genetic associations with shoulder impingement syndrome; (**B**) Forest plot of the causal effects of single nucleotide polymorphisms associated with rotator cuff injury on shoulder impingement syndrome; (**C**) The results of the leave-one-out method suggested that the exclusion of a single SNP does not have a subversive effect on the final conclusions; (**D**) The funnel plot suggests little heterogeneity in this MR analysis

A total of 22 SNPs with strong correlation with RC injury were screened and were used for this MR analysis. The F value of the 22 SNPs included in the MR analysis were all greater than 10 (Supplementary file 1). The result of the IVW method showed a causal association between RC injury and SIS (OR = 1.189, *P* = 0.0059). The results of MR Egger (OR = 1.236, *P* = 0.2013) method, weighted median method (OR = 1.097, *P* = 0.2428) and weighted mode method (OR = 1.013, *P* = 0.930) suggests that the causal association between RC injury and SIS was not statistically significant (Figs. 2 and 3A and B; Table 1). Considering the higher accuracy of the IVW method, the MR results support the causal correlation between RC injury and SIS.

**Table 1:** Two sample MR results of rotator cuff injury on shoulder impingement syndrome

a Results from two sample MR									
method	nsnp				b		se	pval	
MR Egger	22				0.212		0.160	0.201	
Weighted median	22				0.092		0.076	0.227	
Inverse variance weighted	22				0.173		0.063	0.006	
Weighted mode	22				0.013		0.139	0.926	
**b Heterogeneity tests (Cochran’s Q test)**									
**method**			**Q**			**Q_df**			**Q_pval**
MR Egger			29.4			20			0.080
Inverse variance weighted			29.5			21			0.102
**c. Test for directional horizontal pleiotropy (MR-Egger regression).**									
**egger_intercept**		**se**							**pval**
-0.006		0.022							0.794
**d. Test that the exposure is upstream of the outcome.**									
**snp_r2.exposure**				**snp_r2.outcome**		**correct_causal_direction**			**steiger_pval**
0				0		TRUE			0.939

Note R^2^ values are approximate

The results of Cochran’s Q test showed (Table 1) that there was no significant heterogeneity among the IVs included in the MR analysis. The results of the leave-one-out method suggested (Fig. 3C) that the exclusion of a single SNP does not have a subversive effect on the final conclusions. The funnel plot method results (Fig. 3D) also showed low heterogeneity in this MR analysis. MR-Egger regression showed no horizontal pleiotropy in the IVs analysed in this MR analysis (Egger regression intercept = -0.006, Standard error = 0.022, Directionality P value = 0.794).

### Mendelian randomization results of shoulder impingement syndrome on the risk of rotator cuff injury

**Fig. 4:**
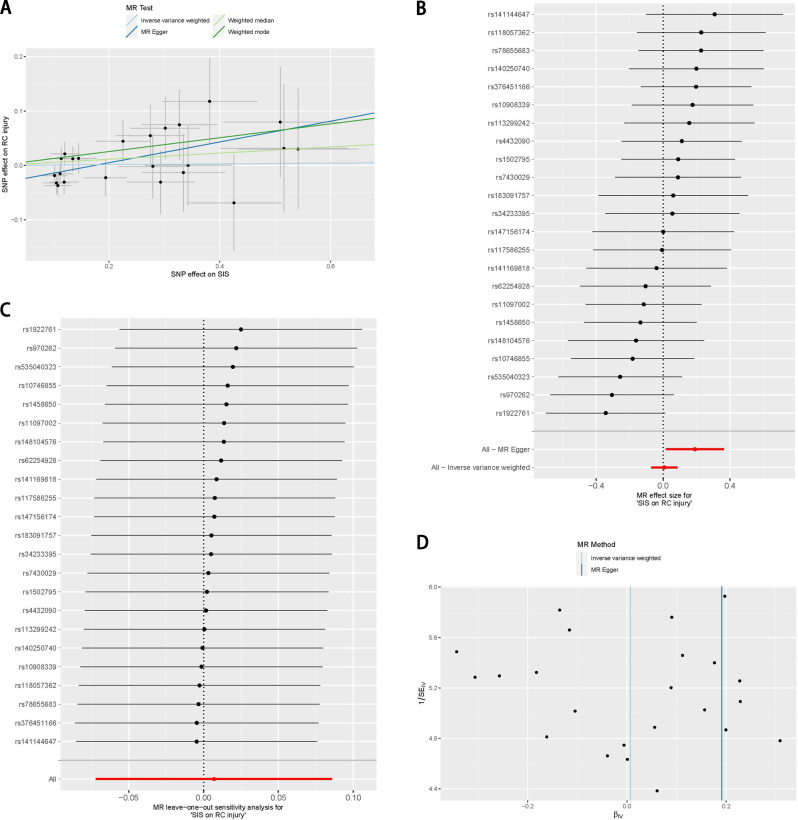
The putative causal effect of shoulder impingement syndrome on rotator cuff injury. (**A**) Scatter plots of genetic associations with shoulder impingement syndrome against the genetic associations with rotator cuff injury; (**B**) Forest plot of the causal effects of single nucleotide polymorphisms associated with shoulder impingement syndrome on rotator cuff injury; (**C**) The results of the exclusion method showed that the exclusion of a single SNP can have a subversive effect on the final conclusions; (**D**) The funnel plot shows significant heterogeneity of MR Egger methods in this MR analysis

Twenty-three SNPs associated with SIS were included in this MR analysis. The F values of all SNPs were greater than 10 (Supplementary file 2). The MR analysis of the method of IVW (OR = 1.007, *P* = 0.862), weighted median (OR = 1.058, *P* = 0.310), and weighted mode (OR = 1.136, *P* = 0.315) showed that there is no causal relationship between SIS and RC injury, and MR Egger (OR = 1.211, *P* = 0.042) showed a causal association between SIS and RC injury (Figs. 2 and 4A and B; Table 2). Considering the accuracy of the IVW method, the credibility of the results is in doubt.

**Table 2:** Two sample MR results of shoulder impingement syndrome on rotator cuff injury

a. Results from two sample MR.								
method			nsnp	b		se		pval
MR Egger			23	0.191		0.088		0.042
Weighted median			23	0.057		0.058		0.328
Inverse variance weighted			23	0.007		0.040		0.862
Weighted mode			23	0.127		0.120		0.299
**b. Heterogeneity tests (Cochran’s Q test).**								
**method**			**Q**	**Q_df**			**Q_pval**	
MR Egger			15.2	21			0.810	
Inverse variance weighted			20.7	22			0.538	
**c. Test for directional horizontal pleiotropy (MR-Egger regression)**								
**egger_intercept**			**se**		**pval**			
-0.033			0.014		0.029			
**d. Test that the exposure is upstream of the outcome**								
**snp_r2.exposure**	**snp_r2.outcome**	**correct_causal_direction**					**steiger_pval**	
0	0	TRUE					0.85	

Note R^2^ values are approximate

The results of Cochran’s Q test showed (Table 2) that there was no significant heterogeneity among the IVs included in the MR analysis. The results of the leave-one-out method suggested (Fig. 4C) that the exclusion of a single SNP have a subversive effect on the final conclusions. The funnel plot method results (Fig. 4D) showed low heterogeneity in this MR analysis. MR-Egger regression showed significant horizontal pleiotropy in the IVs analysed in this MR analysis (Egger regression intercept = -0.0033, Standard error = 0.014, Directionality P value = 0.029). Based on the above results, the results of MR analysis were unreliable that SIS is a risk factor for RC injury.

## Discussion

Since Neer’s first description of SIS in 1972 [9], researchers have relentlessly investigated the mechanisms of its pathogenesis. Based on the theory put forward by Neer that SIS causes RC injury and shoulder pain [9], anterior acromioplasty is widely practised for the treatment of SIS [18–20]. However, the emergence of the intrinsic theory and the results of an increasing number of clinical studies are challenging it [11]. Haahr conducted an RCT comparing the prognostic difference between physiotherapy and surgical treatment for patients with SIS, and the results of the study showed no significant prognostic difference between the two ways [36]. A similar study was done by Ketola, whose findings were similar to Haahr [22]. These studies imply that anterior acromioplasty surgery based on the extrinsic theory may be unhelpful for the progression of SIS. However, the results of the study may be biased due to differences in the quality of the study, follow-up, and type of lesion. The RCT conducted by Rahme has shown that surgical treatment of SIS is more effective than conservative treatment [37]. That’s a completely contradictory conclusion. This confusion in treatment due to differences in theories of pathogenesis has always existed in the treatment of patients with SIS. Therefore, the exploration of its pathogenesis can help to standardise the treatment of SIS.

Our study explored the causal relationship between RC injury and SIS by using MR methods. SNPs with strong correlation with RC injury and SIS were screened as IVs based on GWAS sources. Based on the results of the four MR analysis methods (IVW, MR Egger, Weighted median, and Weighted mode), we found that there may be a causal relationship between RC injury and SIS. The results of MR analysis do not support that SIS is a risk factor for RC injury, and heterogeneity analysis indicated that reverse MR analysis had significant heterogeneity. Therefore, this analysis cannot prove whether SIS is the cause of RC injury. In order to further clarify the causal relationship between the two, we have also tried to use other different datasets for analysis, but the results often show high heterogeneity. This is the biggest deficiency in our research, and it is also the direction that we need to make further efforts in the future. Synthesize the above conclusions, the results of our study suggest that RC injury is causal factor for SIS. This result actually supports the intrinsic theory.

For the painful symptoms of SIS, which are the most prominent disease feature, the extrinsic theory suggests that mechanical damage to the tendon is the primary cause of pain. In fact, recent studies have shown that neuropathic pain as well as central pain may also be involved in SIS pain in addition to injurious pain [38]. This also means that mechanical damage is not the entire cause of painful symptoms in SIS. These studies seem to support the intrinsic theory from another direction, but there is still a lack of sufficient evidence.

In our study, there are some merits. Firstly, we demonstrated a causal relationship between RC injury and SIS by MR analysis. This is the first MR analysis to explore the causal relationship between RC injury and SIS. The study helps us to better define the pathogenesis of SIS, which may contribute to the treatment of SIS in the future. Secondly, the rigorous screening criteria of our SNPs and the evaluation of heterogeneity and horizontal pleiotropy mean that the MR analysis is highly reliable. Of course, there are some shortcomings in our study. Firstly, the participants in this study are all European and may not fully represent the wider population. Secondly, there is heterogeneity in the reverse MR relationship analysis in our study, so it is difficult to further explain whether SIS is the cause of RC injury. This is also the direction that we need to make further efforts in the future. Moreover, there may be duplication of participants in the two samples, which may also affect the final result of MR analysis. This is also the direction that we need to make further efforts to improve in the future.

## Conclusion

Our two-sample MR analysis showed a causal relationship between RC injury and SIS. But the reverse causal relationship between the two still needs further exploration. This finding has important implications for improving our understanding of the complex relationship between RC injury and SIS. This conclusion may have important positive implications for the management of patients with shoulder pain in the future.

### Electronic supplementary material

Below is the link to the electronic supplementary material.

Supplementary Material 1

## Data Availability

The study’s original contributions are contained in the article/supplementary material. Data for this study were obtained from the IEU Open GWAS project (https://gwas.mrcieu.ac.uk/) and GWAS catalog (https://www.ebi.ac.uk/gwas/). Any additional questions should be forwarded to the corresponding authors.
