# Supplementary material for: Exploration of causal relationship between shoulder impingement syndrome and rotator cuff injury: a bidirectional mendelian randomization
Source: BMC Musculoskelet Disord. 2024 Aug 19;25:649. doi: 10.1186/s12891-024-07556-1 (PMC11331745; doi:10.1186/s12891-024-07556-1)
Supplement: Supplementary file 1 — Supplementary Material 1 [file 12891_2024_7556_MOESM1_ESM.docx]

**Caption of the supplementary files**

***Supplementary file 1. MR results of RC injury in SIS.***

1) SNPs information related to RC injury

2) MR harmonise result information of SNPs related to RC injury

3) MR pleiotropy test result

4) F statistic results of SNPs related to RC injury

***Supplementary file 2. MR results of SIS in RC injury.***

1) SNPs information related to SIS

2) MR harmonise result information of SNPs related to SIS

3) MR pleiotropy test result

4) F statistic results of SNPs related to SIS
